# Prediction of spacer-α6 complex: a novel insight into binding of ADAMTS13 with A2 domain of von Willebrand factor under forces

**DOI:** 10.1038/s41598-018-24212-6

**Published:** 2018-04-10

**Authors:** Xiang Fang, Jiangguo Lin, Ying Fang, Jianhua Wu

**Affiliations:** 1 Institute of Biomechanics/School of Bioscience and Bioengineering, South China University of Technology, Guangzhou, 510006 China; 2 Guangzhou Institute of Pediatrics, Guangzhou Women and Children’s Medical Center, Guangzhou, 510623 China

## Abstract

Force-regulated cleavage of A2 domain of von Willebrand factor (vWF) by ADAMTS13 is a key event in preventing thrombotic thrombocytopenic purpura (TTP). Recognition and cleavage depend on cooperative and modular contacts between several ADAMTS13 subdomains and discrete segments of vWF A2 domain. Spacer domain of ADAMTS13 contains an important exosite interacting with α6 helix of unfold A2 domain, but it remains unclear whether stretching of α6 regulates binding to spacer. To understand the molecular mechanism underlying the interactions between spacer and α6 under stretching, we successfully predicted spacer-α6 complex by a novel computer strategy combined the steered molecular dynamics (SMD) and flexible docking techniques. This strategy included three steps: (1) constant-velocity SMD simulation of α6; (2) zero-velocity SMD simulations of α6, and (3) flexible dockings of α6 to spacer. In our spacer-α6 complex model, 13 key residues, six in α6 and seven in spacer, were identified. Our data demonstrated a biphasic extension-regulated binding of α6 to spacer. The binding strength of the complex increased with α6 extension until it reaches its optimum of 0.25 nm, and then decreased as α6 extension further increased, meaning that spacer is in favor to binding with a partially extended α6, which may contribute to the optimal contact and proteolysis. Changes of interface area and intermolecular salt bridge may serve as the molecular basis for this characteristic. These findings provide a novel insight into mechano-chemical regulation on interaction between ADAMTS13 and vWF A2 domain under forces.

## Introduction

As a multimeric plasma glycoprotein, von Willebrand factor (vWF) is synthesized in vascular endothelial cells and megakaryocytes, secreted constitutively or stored in granules as “ultra large” multimers (UL-vWF)^1,2^. Upon stimulation, the UL-vWF is secreted and immediately anchored onto the cell surfaces^3^, playing a key role in physiological hemostasis and pathological thrombosis through spontaneously forming high-strength bonds with glycoprotein Ib-IX-V complex^4,5^. Prothrombotic UL-vWF is specifically cleaved by the enzyme ADAMTS13 (A Disintegrin and Metalloprotease with thrombospondin motifs-13), converting the ultra-large multimer into smaller and less adhesive one^6^. Congenital or acquired deficiency of ADAMTS13 causes the accumulation of the UL-vWF multimers^7^ and thereby leads to thrombotic thrombocytopenic purpura (TTP), a life-threatening disease characterized by thrombocytopenia, hemolytic anemia, neurological and renal manifestations, as well as fever^8,9^. Conversely, excessive cleavage leads to von Willebrand disease (vWD) type 2A, a qualitative vWD variant characterized by the absence of large vWF multimers^10^.

**Figure 1:**
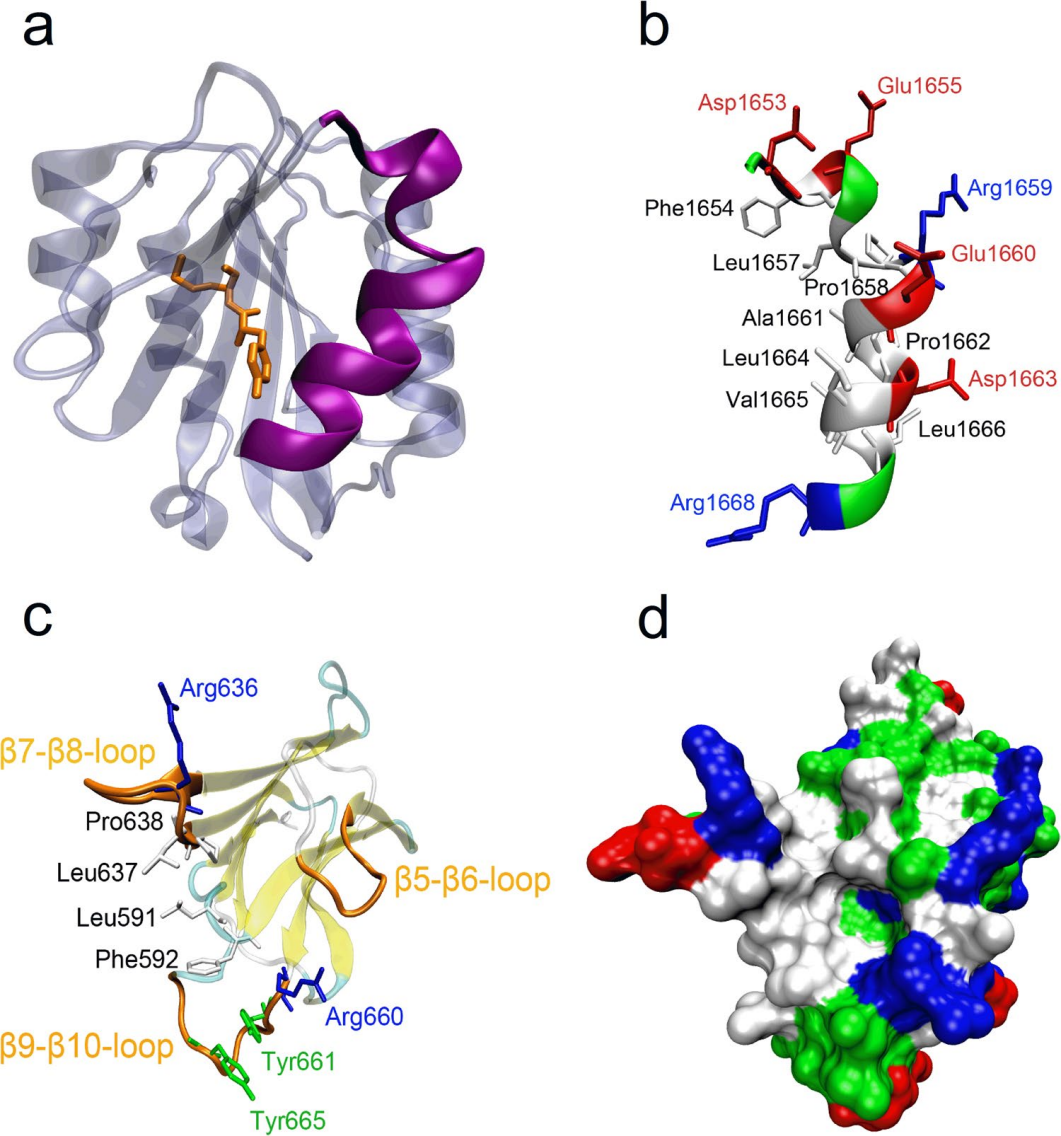
Structure of the α6 helix of vWF-A2 and spacer domain of ADAMTS13. (**a**) Structure of vWF-A2 domain. The α6 helix is colored according to polarity of the residues, while the remainder of the protein subunit (iceblue) is shown in transparent cartoon representation. ADAMTS13 cleavage site residues (Tyr1605 and Met1606) are shown as orange sticks. (**b**) Close-up view of the α6 helix. Hydrophobic residues, acidic residues and basic residues are shown as white, red and blue sticks, respectively. (**c**) Structure of ADAMTS13-spacer domain. The β5-β6-loop, β7-β8-loop and β9-β10-loop are highlighted in orange, while the remainder of the protein subunit is shown in transparent cartoon representation. Uncharged polar residues are shown in green; other residues are colored as in (**b** and **d**) Surface representation of the spacer domain. Residues are colored as in (**b**).

ADAMTS13 consists of a metalloprotease domain (M), a disintegrin-like domain (D), a thrombospondin type 1 repeat (TSP1, T), a Cys-rich domain (C), a spacer domain (S), seven additional TSP1 repeats and two unique CUB (complement components C1r/C1s, urchin epidermal growth factor, and bone morphogenic protein-1) domains^11^. ADAMTS13 specifically cleaves the peptide bond Tyr1605-Met1606 buried in the hydrophobic core of the vWF A2 domain, which adopts a Rossman fold with six central β-strands flanked by five α-helices^12^ (Fig. 1a). However, cleavage cannot occur unless A2 has been unfolded, either by tensile force^13–15^ or by chaotropic agents^16^, to induce the exposure of the scissile bond. The single metalloprotease domain is unable to cleave this peptide bond, while the truncated mutant MDTCS is considered to be the minimum unit that possesses comparable proteolytic activity as wild type^17–20^. However, the proximal carboxyl-terminal domains of ADAMTS13 are also required for recognition and cleavage^21^. The cooperative and modular contacts between several ADAMTS13 domains and discrete segments of A2 contribute to substrate specificity^22^.

The ADAMTS13 spacer domain, which folds into a single globular domain with 10 β-strands in a jelly-roll topology (Fig. 1c), contains an important exosite^23^, interacting with the amphipathic α6 helix of A2^22^ (Fig. 1b) and being essential for the ADAMTS13 activity^17^. It was demonstrated that deleting the spacer domain from ADAMTS13 or deleting the α6 helix from vWF A2 reduced the rate of cleavage approximately 20-fold^19^. The α6 helix, the Asp1653-Arg1668 segment located in the C-terminal of A2 (Fig. 1a and b), consists of eight hydrophilic (six charged and two uncharged) residues and eight hydrophobic residues^12^. The importance of charged residues were shown as mutating Asp1653 and Asp1663 to Ala significantly reduced the cleavage towards the substrate peptide, and the Glu1655Ala mutation slightly increased cleavage^24^. The vWF-binding exosite-3 in ADAMTS13, a hydrophobic cluster rimmed with arginine residues (Arg636 and Arg660) (Fig. 1c and d), is constructed by the solvent-accessible Tyr661 and Tyr665 residues located at the distal β9-β10-loop, together with Pro590, Leu591, Phe592, Leu637 and Pro638, in the spacer domain. The tyrosine residues (Tyr661 and Tyr665) in exosite-3 were indicated to be critical for proteolysis towards vWF A2 peptide^23,25^. In forming a contact with ADAMTS13, the α6 helix in A2 might orientate its hydrophobic residues towards the exosite-3^23,25^.

Binding of the spacer of ADAMTS13 to the α6 helix in A2 may be a key event in the recognition of ADAMTS13 towards vWF. This event should be force-dependent because it occurs after the unfolding of A2 in flow^23,25^. However, the underlying molecular mechanism of the event remains unclear due to the lack of crystal structure of spacer-α6 complex. Although the structures of A2 and N-terminal DTCS domains of ADAMTS13 were crystalized respectively^12,23^, it is hard to provide the insights into interaction of spacer with the α6 helix stretched under forces. Besides, the stretched α6 helix under forces provides another barrier in building a computational model of the spacer-α6 complex, although protein-protein docking can predict complex structure on the basis of its known components^26^.

To uncover the molecular basis underlying the interactions between the spacer and the α6 helix under forces, we herein predict the conformation of spacer bound with the stretched α6 helix through a novel computer strategy combined steered molecular dynamics (SMD) simulations^27,28^ with the flexible molecular docking^27,28^. In this hybrid method different from the classical ensemble docking methods^27,28^, the stable conformations of α6 under tensile forces were generated by SMD simulations first and then used to construct the spacer-α6 complex model through flexible docking, mimicking the induced-fit process of binding of spacer to stretched α6 helix. With this spacer-α6 complex model, we examined how the extension of α6 affects its binding strength with spacer. The results demonstrated that the binding of spacer to stretched α6 was biphasic extension-dependent, that is, the spacer is in favor of moderately but not excessively stretched A2. This new finding should be contributed to better understanding of force-dependent recognition and cleavage of A2 by ADMATS13, and the present computer strategy would serve as a novel tool in the structure-function research of other mechano-sensitive proteins.

## Results

### Unfolding α6 by constant-velocity SMD

**Figure 2:**
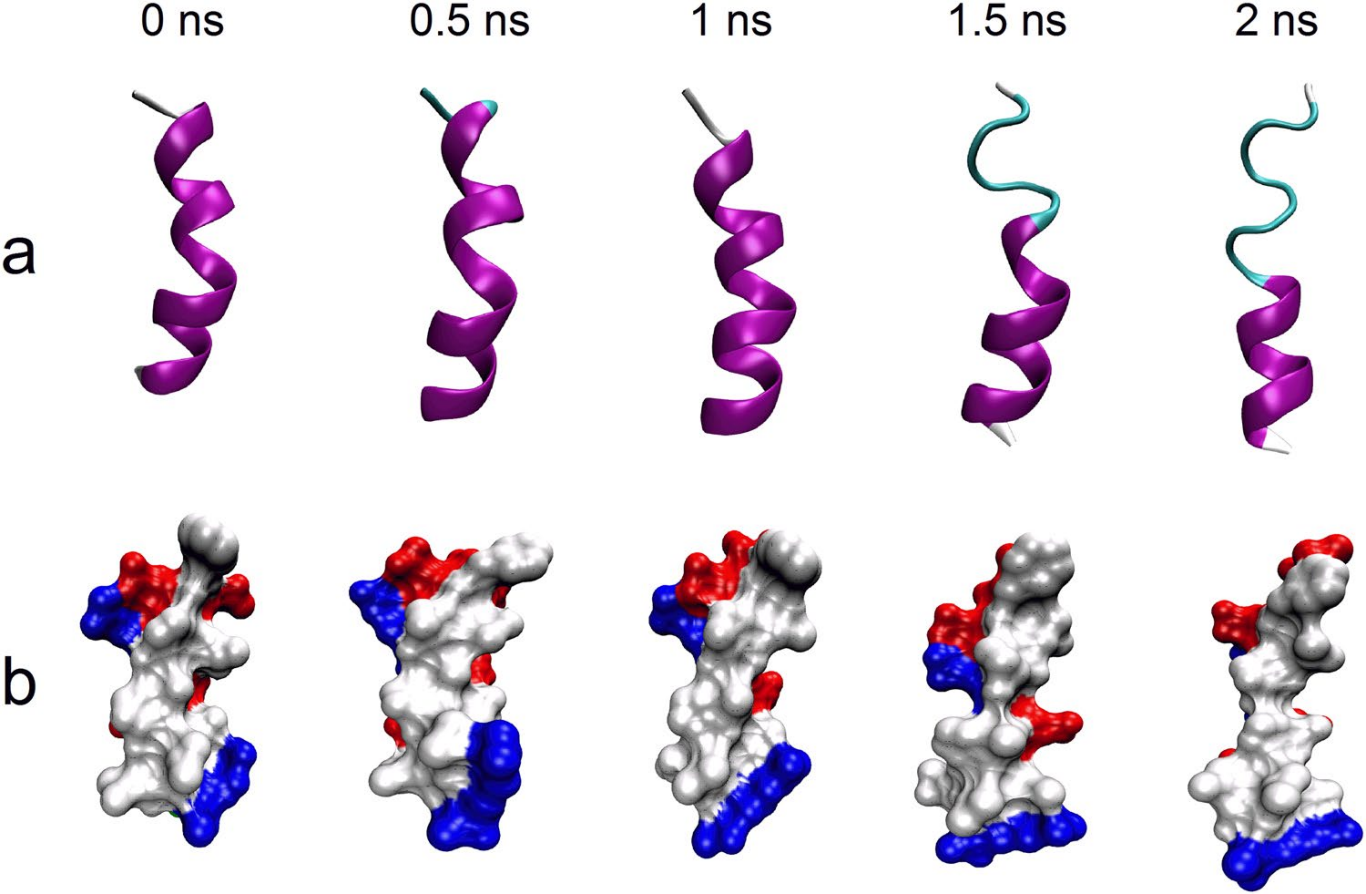
Snapshots of α6 in constant-velocity SMD simulation at indicated times. (**a**) Newcartoon representation of α6. (**c**) Surface representation of α6. Hydrophobic, acidic residues and basic residues are shown in white, red and blue, respectively.

Binding of spacer to α6 requires unfolding of vWF-A2 domain^23,29^. This observation prompted us to hypothesize that unfolding (or extension) of α6 may be a key event in binding with spacer. To test this hypothesis, we suggested a novel computer strategy combined with SMD simulation and flexible docking to construct a model of spacer bound with extended α6 (Materials and Methods). For gaining conformations of α6 with various extensions, we here firstly simulated unfolding of α6 by constant-velocity(cv) SMD simulation with pulling velocity of 0.5 nm/ns. Snapshots of five intermediates at different time points were shown here (Fig. 2a). The unfolding status at N-terminal (Asp1653-Arg1659), indicating that the C-terminal segment Glu1660-Arg1668 is more stable. Structural analysis revealed that the segment Asp1653-Leu1657 forms a 3_10-_helix (Fig. 1b), which confers a lower stability than the α-helix formed by the segment Arg1659-Arg1668. Furthermore, steering α6 not only increases its length (from 2.089 nm to 2.921 nm) but also changes the shape of hydrophobic surface, which was gradually elongated (Fig. 2b, white region). It has been proposed that α6 binds to spacer with hydrophobic residues^23^. Changing the shape of hydrophobic surface also alters the position of hydrophobic residues, and thus may affect the binding mode and even binding strength with spacer.

### Relaxing conformation of α6 by zero-velocity SMD

Although cv-SMD is necessary to unfold α6, the intermediates are not suitable for docking. This is because of that, the limitation of computational resources here leads to a pulling speed of 0.5 nm/ns, which is much higher than that in AFM (atomic force microscopy)^13^ and laser tweezers experiments^12,30^. Thus, the transient conformational change of α6 in cv-SMD simulation may be localized. And, due to the soft spring used in the simulation, the SMD atom often lag behind the dummy atom, and extensions of α6 were often less than the expected values (e.g., at 2 ns, the transient extension was 0.832 nm but not 1 nm as expected). Therefore, further simulations were required to relax the stretched α6 conformations sampled from cv-SMD simulation.

**Figure 3:**
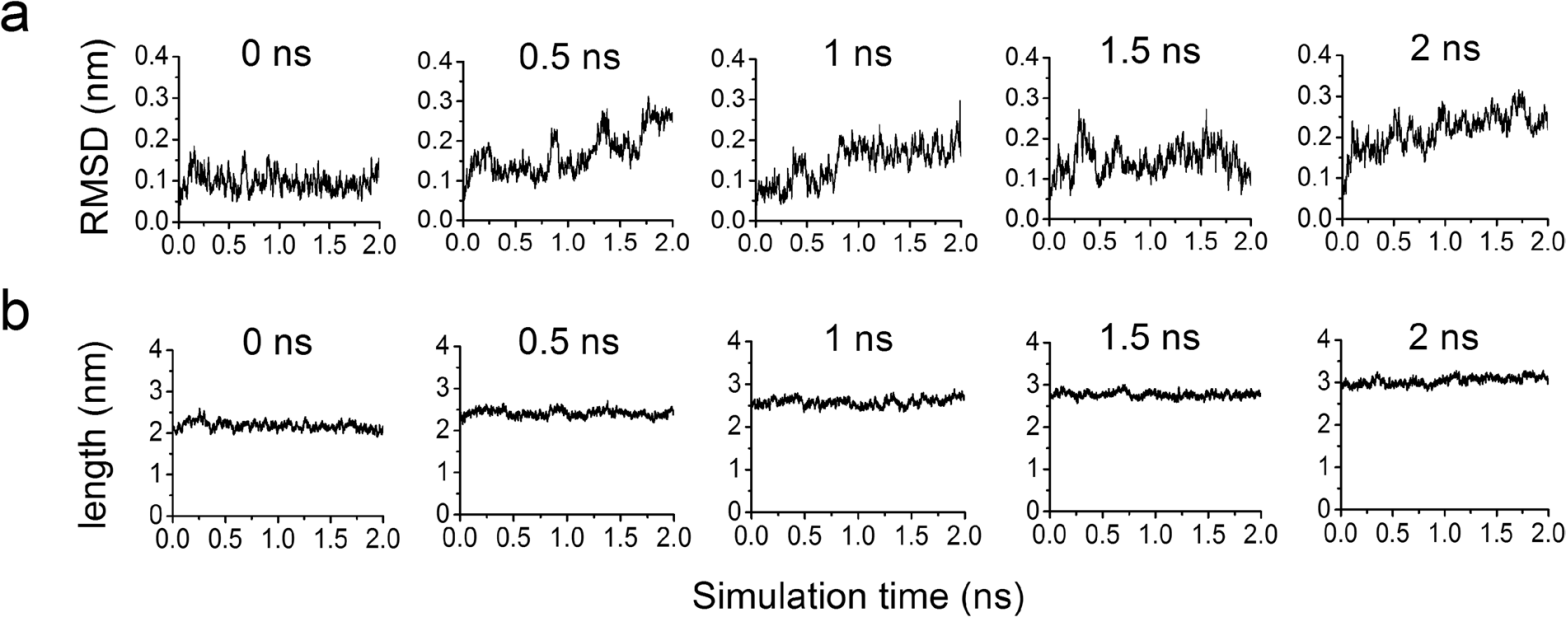
Zero-velocity SMD simulations of α6. (**a**) RMSD of heavy atoms of α6. (**b**) Length of α6.

To generate feasible conformations for docking, five intermediates of α6, so called as the first, second, third, fourth and fifth structures, were taken from cv-SMD simulation at 0.0, 0.5, 1.0, 1.5 and 2.0 ns, respectively, and further subjected to zero-velocity(zv) SMD simulation. Compared with traditional equilibration or free dynamics simulation, zv-SMD retained original steering force of cv-SMD and relaxed the conformation of α6 under constraint. Conformational change could be rearranged within α6, which could also achieve the expected length. To illustrate the stability of α6 in zv-SMD, RMSD of heavy atoms was calculated (Fig. 3a). The 1^st^ and 3^th^ structures are more stable than the others, because their RMSD time-courses had their respective “pseudoplateaus” over time from ~1000 to ~1912 ps or from ~864 to ~1924 ps. The 2^nd^, 4^th^ and 5^th^ structures almost remained steady in ~282 to 802 ps, ~724–1502 ps and ~914–1640 ps, respectively, during zv-SMD simulations. However, the extension of α6 remain almost constant during zv-SMD simulation (Fig. 3b). The obvious atom fluctuation involved in each structure in zv-SMD simulation (Fig. 3a), and mainly came from non-bonded energy but not bond, angle, dihedral and improper energies (Data not shown). Three transient relaxed conformations were randomly selected from each structure in the “pseudoplateaus” of zv-SMD simulation for docking, with extension constraint that the extension difference between two sampled conformations was in the range of ± 0.04 nm (Table 1).

**Table 1:** Length and extension of extended α6 bound with or without spacer*.

Δ_E_ (nm)	L_F_ (nm)	L_B_ (nm)	Δ_S_ (nm)	Δ_M_ (nm)
0.00	2.058	1.916	0.142	0.143
	2.101	1.961	0.14	
	2.076	1.929	0.147	
0.25	2.332	2.392	−0.06	0.097
	2.332	2.272	0.06	
	2.358	2.066	0.292	
0.50	2.57	2.465	0.105	0.010
	2.603	2.627	−0.024	
	2.594	2.646	−0.052	
0.75	2.857	2.715	0.142	0.137
	2.799	2.675	0.124	
	2.837	2.691	0.146	
1.00	3.121	2.852	0.269	0.198
	3.058	2.89	0.168	
	3.122	2.964	0.158	

^*^Where, Δ_E_ indicates the extension of α6 pose sampled from cv-SMD simulation, L_F_ and L_B_ express the lengths of the extended α6 before and after docking, respectively, Δ_S_ (=L_F_ − L_B_) is the retraction length of bound α6 in comparison with its free extended pose, and Δ_M_ is the mean of retraction lengths of three sampled poses for a given Δ_E_. The length of α6 after equilibration without stretching is 2.09 nm.

### Well-constructed models of spacer bound with extended α6 via flexible docking

**Figure 4:**
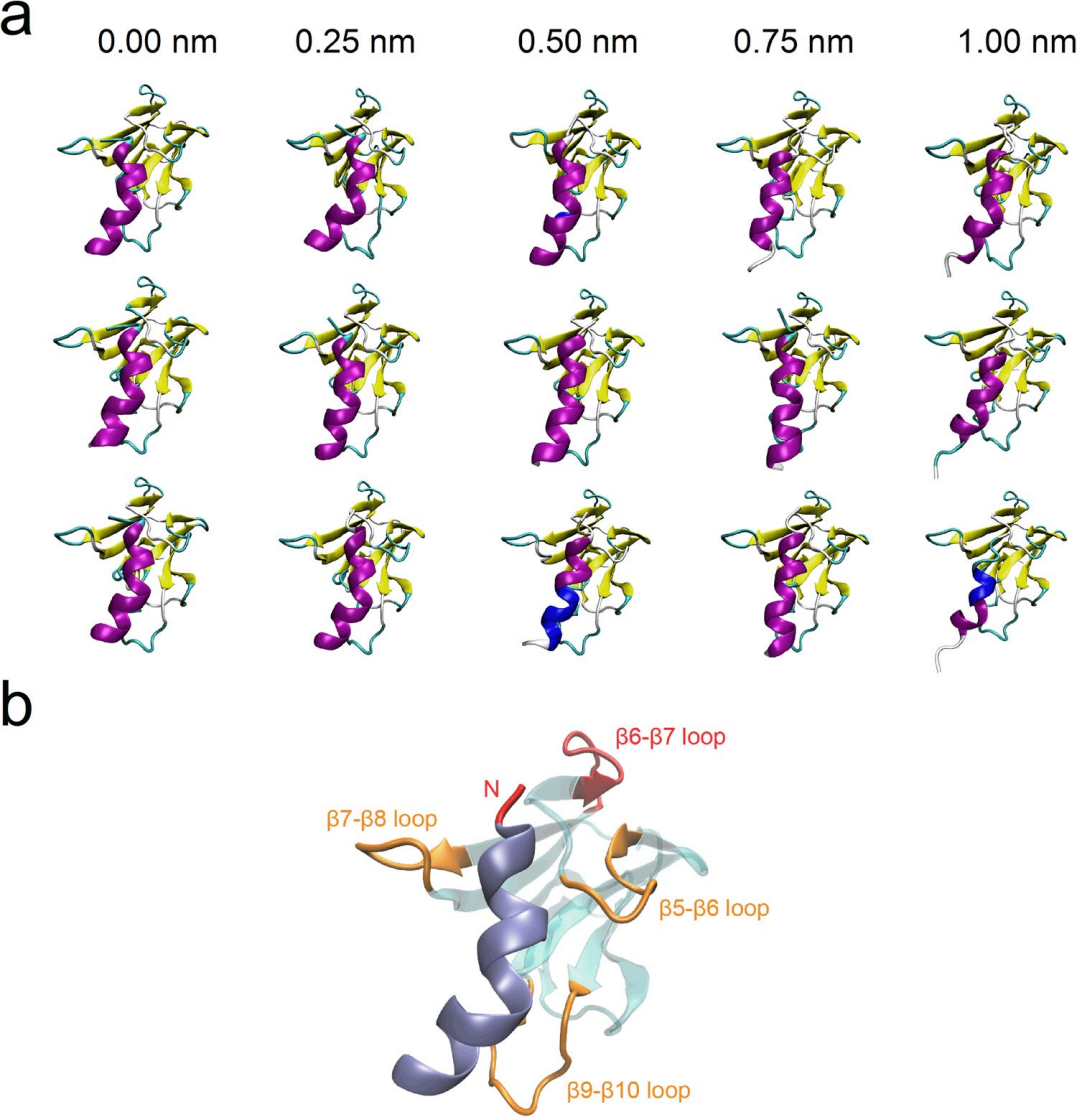
Spacer-α6 complex models. (**a**) Models with different extensions of α6. Models are shown in cartoon representations. Spacer is colored as in Fig. 1c; α6 is colored according to its secondary structure, as purple (α-helix) or blue (3_10_-helix), respectively. The three models in each column come from docking of spacer with three different starting poses of extended α6 of same extension, such as zero, 0.25, 0.5, 0.75 or 1 nm. (**b**) The binding site of α6 on spacer. Spacer is shown in transparent cyan with three loops β5-β6, β7-β8, β9-β10 colored with orange. The β6-β7 loop is highlighted with red. α6 helix is shown in ice blue, with the N-terminal colored with red.

To build the spacer-α6 complex model, flexible docking of extended α6 to spacer was performed via HADDOCK^31^. From all generated models of the spacer bound with each extended α6 structure selected randomly in zv-SMD simulation, one was considered to be proper (Table S1–5), suggested by the correct orientation between N-termini of spacer and α6, proper relative position between the two molecules, and a lowest HADDOCK score without completely losing of the helical structure integrity of α6 (Materials and Methods). Fifteen complex models (Fig. 4a) in total were selected. The results demonstrated that there was a moderate extension difference (<0.20 nm) between the initial free stretched α6 and its bound pose (Table 1). However, the bound α6 still remained a higher helical structure integrity (Fig. 4), even though it seemed that there was a partially retracting process for an extended α6 in docking. The relative position between the spacer and α6 became bad if the tensile elongation of α6 was over 1 nm, meaning the over-stretched α6 was not in favor of binding to the spacer. Of the three complex models of the spacer bound to each extended α6 structure (Fig. 4a), each exhibited a correct orientation of α6, because it directed the N-terminal of α6 toward the apical β6-β7 loop of spacer (the boundary with N-terminal Cys-rich domain) (Fig. 4b), and illustrated that the binding site of α6 on the spacer was located at the β5-β6-loop, β7-β8-loop and β9-β10-loop, where the latter two loops had been identified to contain the key residues for proteolysis^23^. Besides, in each model (Fig. 4), the hydrophobic surface of the spacer and α6 faced with each other to form a contact, as suggested by Gao *et al*.^22^.

### Moderately Stretching induces α6 in favor of binding with the spacer

**Figure 5:**
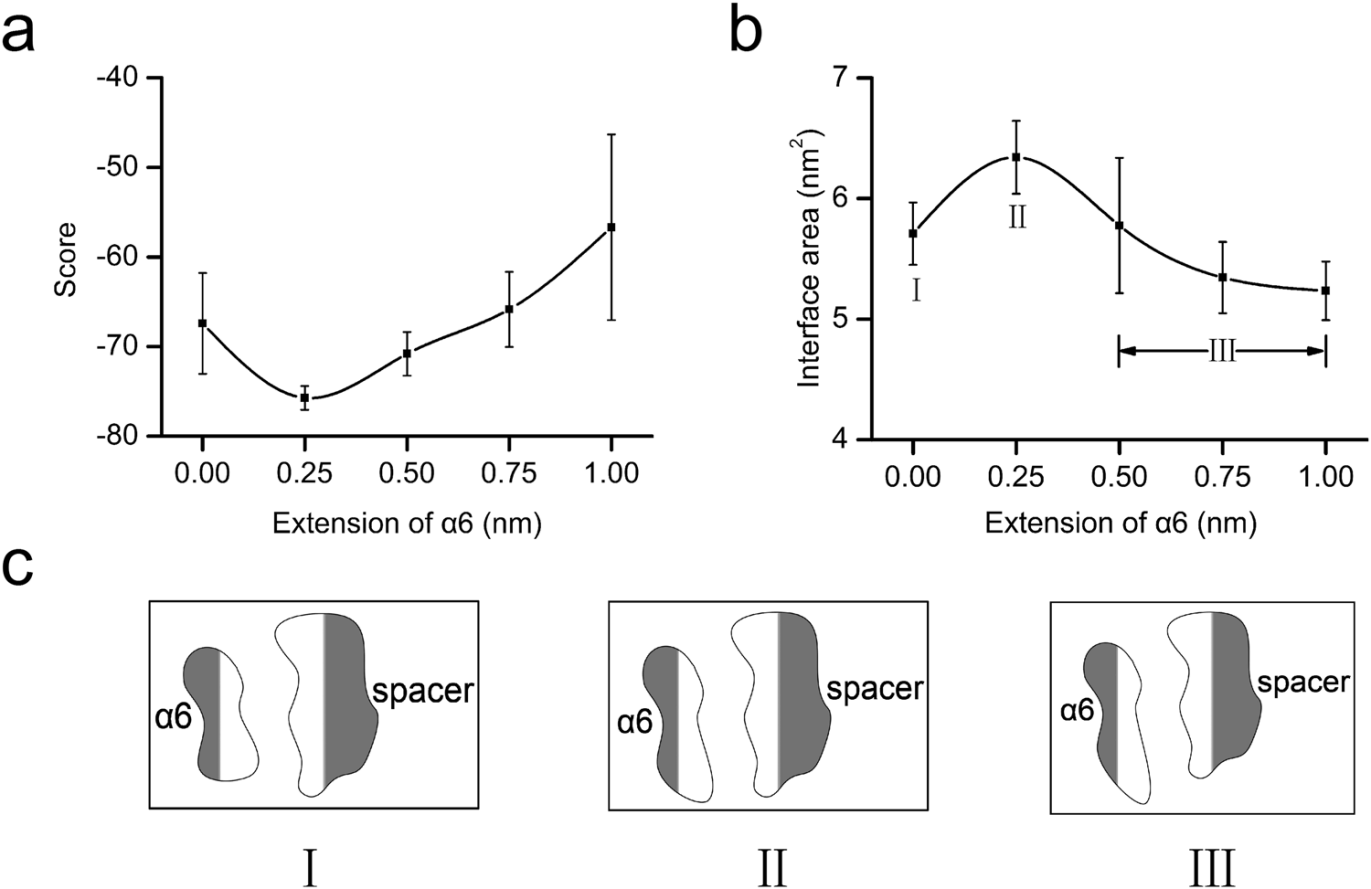
Extension-dependent binding strength of spacer-α6 complexes. (**a**) Score of docking complexes is plotted versus the extension of α6. The lower the score, the stronger the binding of α6 to spacer; (**b**) Interface area of complex models is plotted versus the extension of α6; and (**c**) Schematics for the induced-fit process of binding of spacer to stretched α6. Where, the light and dark regions indicate the hydrophobic and hydrophilic surfaces, respectively, and the label I,II and III express the three stages, respectively, for the enhanced, the best and the weakened binding of spacer to α6 under tensile force. All data in (**a**) and (**b**) are presented as mean ± SD of 3 complexes.

Tension may enhance binding of spacer to the moderately stretched α6. To verify this hypothesis, we here examined the strength of binding of spacer to α6 with extensions ranging from zero to 1 nm. As a weighted sum of van der Waals, electrostatic, desolvation and ambiguous interaction restraints energies^32^, HADDOCK score should reflect the binding strength between the spacer and α6. Plot of HADDOCK score against extension of α6 illustrated that the HADDOCK score decreased first and then increased with extension of α6, and the minimum occurred at extension of about 0.25 nm (Fig. 5a). It suggested that stretching enhanced formation of spacer-α6 complex, until extension of α6 reached its optimum, at which the interaction energy between the spacer and α6 lied in the minimum level. Over the optimum of extension, potential stored increasingly in α6 might cause an increase of the interaction energy between the spacer and α6, leading to more loosely binding of the spacer with the stretched α6.

Binding of ADAMTS13-DTCS to vWF73 (a segment of vWF-A2, from Asp1596 to Arg1668)^7,23^ suggests an induced-fit mechanism for the ADAMTS13 cleavage^7,23^. This induced-fit mechanism may be demonstrated by that, stretching would make α6 allosteric, and not only change the hydrophobic surface of α6 but also influence matching between the hydrophobic surfaces of the spacer and α6. To verify this, we examined the hydrophobic interaction of the spacer and α6 by the hydrophobic interface area, which was defined as a half of total decrease of solvent accessible surface area (SASA) of the two molecules upon interaction formation^33^. The results indicated that the hydrophobic interface area increased first and then decreased with extension of α6, and, reached its the maximum at the extension optimum of 0.25 nm (Fig. 5b), in consistent with the HADDOCK score (Fig. 5a). This biphasic pattern of the hydrophobic interface area also suggested that binding of the spacer to α6 was promoted first and then attenuated by stretching the α6 peptide.

### The key residues in binding site of the α6-spacer complex

**Figure 6:**
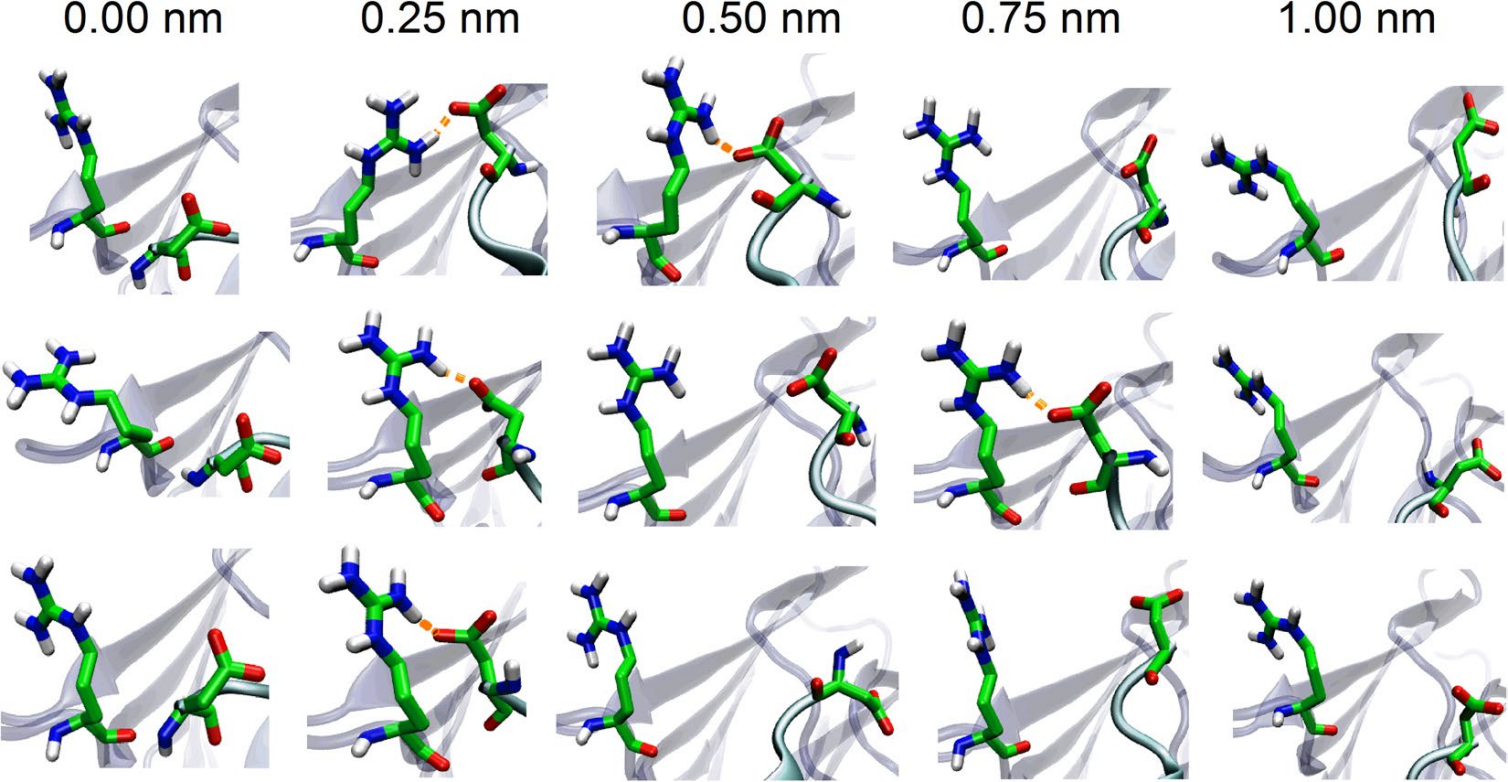
Extension of α6 significantly affects the ion interaction. Salt bridge Asp1653-Arg636 is indicated with orange dotted line. Hydrogen atoms, oxygen atoms, nitrogen atoms and carbon atoms are indicated with white, red, blue and green, respectively. Spacer is shown in cyan transparent cartoon representation; α6 is shown with iceblue opaque cartoon representation.

The residue Asp1653 in α6 was validated as a key residue in substrate cleavage^24^. The reason might come from the crucial role of Asp1653 on binding of the spacer to α6. We found herein that, except the salt bridge between Arg636 in the spacer and Asp1653 in the stretched α6 with tensile elongation of 12%, 24% and 36%, there were no any other hydrogen bonds and/or salt bridges among the fifteen conformations of the spacer-α6 complex (Fig. 6). The salt bridge Arg636-Asp1653 occurred in each complex pose for α6 with the tensile elongation of 11.97%, and just in one as the tensile elongation of α6 was 24% or 36%. These results suggested that stretching promoted first and then impeded the bonding of Arg636 to Asp1653, and the switch point might occur at the optimal extension of 0.25 nm, which was shared with the patterns of both the HADDOCK score and the hydrophobic interface area (Fig. 5a and b). The residue Arg636 surrounded hydrophobic interface (Fig. 1c) of exosite-3 in spacer may be responsible for binding of spacer to α6, especially as α6 was stretched with extension of 0.25 nm.

Eighteen hydrophobic interactions between spacer and α6 at the extension of 0.25 nm (Table 2) were obtained through PIC server (a tool to calculate the interactions by the standard and published criteria as the coordinate set of 3D structure of a protein is given) (http://pic.mbu.iisc.ernet.in/job.html)^34^. Of the hydrophobic residues involved in these interactions, Leu591, Phe592, Tyr661 and Tyr665 in spacer were identified to be crucial^23,25,35^ and would interact with Ala1661, Pro1662, Leu1664 and Val1665 in α6 (Table 2). This result is not only in consistency with previous finding that both Tyr661 and Tyr665 interacted with exosite 1660–1668 within vWF A2 domain^25^ but also in agreement with the significant inhibiting effect of the peptide from Ala1661 to Leu1664^36^. Besides, Leu1657 and Phe1654 in α6 also might responsible for binding of spacer with α6, through interacting with their respective hydrophobic residue clusters (Ile611, Pro638, Leu591 and Leu637; Ile611, Pro638, Leu632 and Val630) in spacer (Table 2). However, we did not observe any interactions formed by other two identified key residues Asp1663^24^ in α6 and Arg660^25,35^ in spacer. It might come from that the conformational space sampled herein was not complete, leading to losing of the constructed complex conformation information; and alternatively, mutating Asp1663 to Ala would change the amphipathy of α6 so that a larger hydrophobic cluster in α6 might form through linking up the surrounding hydrophobic residues (Ala1661, Pro1662, Leu1664, Val1665, Leu1666 and Gln1667) with each other (Fig. 1b), leading to changing of the relative orientation of α6 with spacer.

**Table 2:** Hydrophobic interactions between spacer and α6.

No	Residue pair		No	Residue pair	
	spacer	α6		spacer	α6
1	Leu591	Leu1657	10	Leu637	Leu1657
2	Leu591	Leu1664	11	Leu637	Leu1664
3	Phe592	Ala1661	12	Pro638	Phe1654
4	Phe592	Leu1664	13	Pro638	Leu1657
5	Phe592	Val1665	14	Tyr661	Pro1662
6	Ile611	Phe1654	15	Tyr665	Pro1662
7	Ile611	Leu1657	16	Tyr665	Val1665
8	Val630	Phe1654	17	Tyr665	Leu1666
9	Leu632	Phe1654	18	Leu668	Val1665

## Discussion

Cleavage activity of ADAMTS13 towards vWF A2 domain is highly dependent on the interaction between spacer and α6^19^. However, it remains unclear whether the spacer will bind with a stretched α6 more easily or not as increasing of α6 extension. An obvious hard barrier lies in lack of technique for both crystal structure analysis and computer model of spacer bound with an extended α6. Previous studies on stretch-induced unfolding of A2 domain did not refer to extension-induced change of stretched A2 affinity with ADMATS13 but focused on tensile-induced exposure of binding and cleavage sites of A2 for ADAMTS13^12,13,30^. Again, classical protein-protein docking will encounter barriers in prediction of spacer-α6 complex conformation, because α6 hidden in native A2 domain cannot interact with spacer until A2 is unfolded^19^. Here, we built a model of spacer bound with extended α6 complex through a novel computer strategy, which included three steps: (1) unfolding α6 by cv-SMD; (2) generating stable conformations by zv-SMD and (3) flexible docking of α6 to spacer (Materials and Methods). Different from classical ensemble docking methods that sample consecutive conformations from conventional MD simulations^27,28^, the present method adopts forced-extended conformations with various lengths from zv-SMD simulations, showing a much more capacity for induced-fit mechanism. The flexible docking program HADDOCK allows full structural flexibility for both side chains and backbone^31^, and may outperform the rigid or semi-flexible docking method in simulating local structural adaption.

Interestingly, with our spacer-α6 model, we found that moderate extension of α6 is advantageous for binding, while excessive extension is detrimental (Fig. 5), as it was pointed that ADAMTS13 may prefer a partially unfolded vWF A2 domain to an overextend one^37^. In fact, ADAMTS13-DTCS may accommodate a partially unfolded vWF73, because the length of fully unfolded vWF73 was almost twice the distance (20 nm) between the catalytic site and the exosite-3^23^; Tsai *et al*. reported that pretreatment with 1.25 mol/L guanidine-HCl increases the susceptibility of vWF to ADAMTS13, but a higher concentration causes vWF to become resistant^38^; and Shim *et al*. revealed that platelet-dependent cleavage of vWF exhibited a maximum between 10 and 30 dyn/cm^2^, and declined at higher shear stress^37^. In addition, we recently found that the force-dependent proteolytic efficiency of ADAMTS13 towards vWF-A1A2A3 tridomain was biphasic, and the optimum occurred at wall shear stress of 0.2 dyne/cm^2^ (Data not shown). In addition to spacer domain containing the exosite-3, the proximal N-terminal disintegrin and Cys-rich domains contain the exosite-1 and −2, targeting to the Asp1596-Ile1623 and Ile1642-Gln1652 segments buried in vWF-A2, respectively^22,23^, implying the most efficient positioning and binding might exist between the exosites and respective binding sites in vWF-ADAMTS13 interaction.

**Figure 7:**
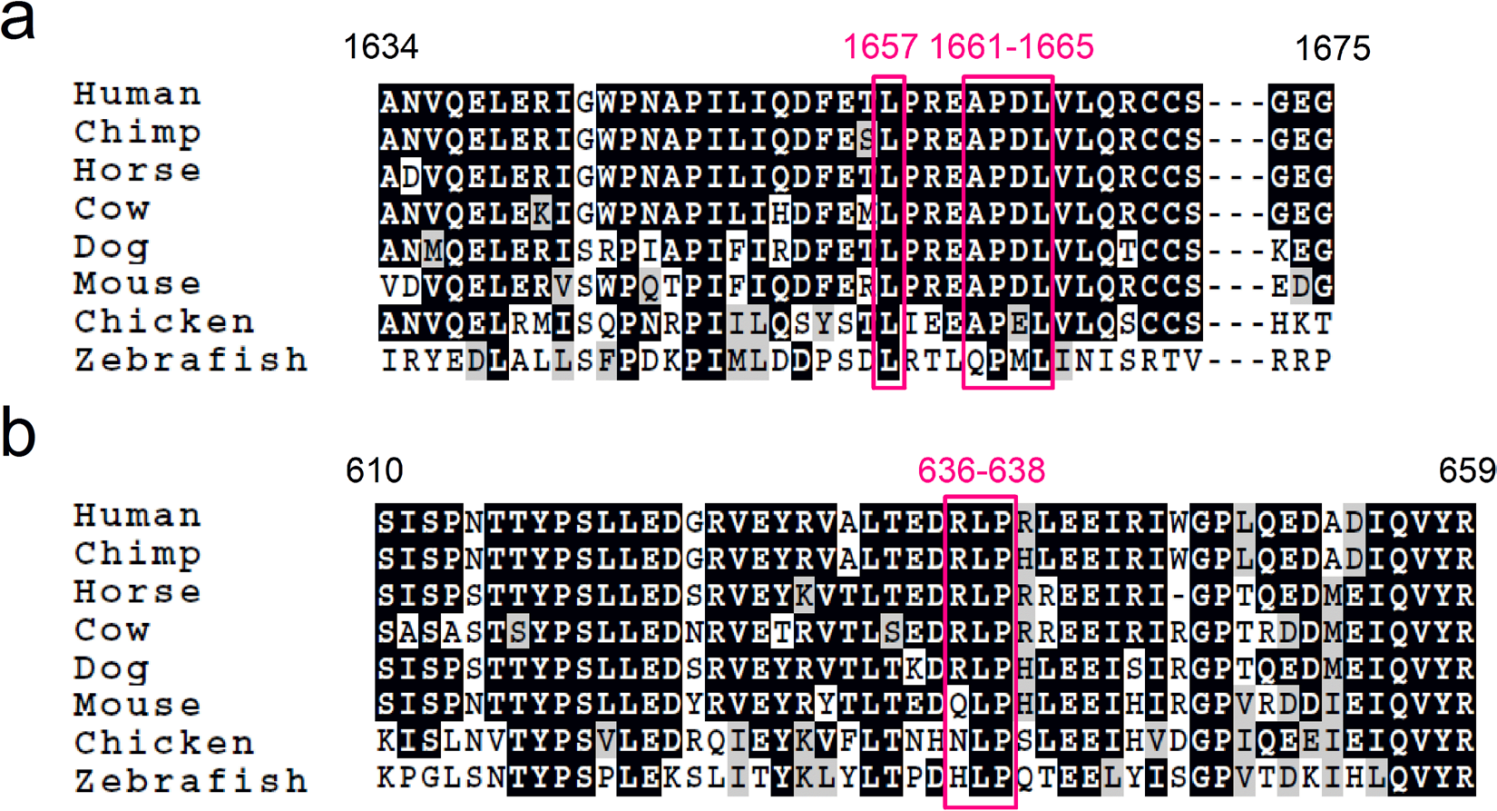
Putative key residues in α6 and spacer. (**a**) Alignment of the vWF-A2 domain from different species. Putative key residues are indicated with pink boxes. (**b**) Alignment of the spacer domain from different species.

Previous experiments have determined some key residues for binding (Column 1 and 3 in Table 3). Through investigating the hydrophobic and ionic interactions with the present complex models, the key residues Asp1653 in α6, and Leu591, Phe592, Tyr661 and Tyr665 in spacer were identified (Column 2 and 4 in Table 3), in consistent with previous findings^23–25,35^. Besides, our data also indicated that Leu1657, Ala1661, Pro1662, Leu1664 and Val1665 in α6, as well as Arg636, Leu637 and Pro638 in spacer are potential key residues. Sequence alignments demonstrated that these residues are highly conserved among different species with an exception of Arg636 (Fig. 7a and b), which is replaced respectively by Gln and Asn in mouse and chicken. We suspect that, in these two species, the salt bridge Asp1653-Arg636 (Fig. 6) is replaced by an H-bond (Asp1653-Gln636 or Asp1653-Asn636). Although previous studies have indicated the importance of Asp1663^24^ and Arg660 by mutation assays^25,35^, we did not observe any interactions formed by Asp1663 in α6 and Arg660 in spacer. The discrepancy might due to the limited sampling space during SMD and docking processes. And, mutating Asp1663 to Ala would significantly modify the amphipathy of α6 through linking its neighboring hydrophobic residues (Ala1661, Pro1662, Leu1664, Val1665, Leu1666 and Gln1667) (Fig. 1b) to form a larger hydrophobic cluster, which may influence the relative orientation with spacer.

**Table 3:** The key residues identified through mutation experiments and predicted with present complex model.

Key Residues in α6		Key Residues in spacer	
Identified	Predicted	Identified	Predicted
Asp1653	Asp1653	Leu591	Leu591
Asp1663	—	Phe592	Phe592
—	Leu1657	—	Arg636
—	Ala1661	—	Leu637
—	Pro1662	—	Pro638
—	Leu1664	Arg660	—
—	Val1665	Tyr661	Tyr661
		Tyr665	Tyr665

Moreover, intrinsically disordered proteins (IDPs), which undergo folding upon binding to their physiological targets^39^, share some similarities with vWF-A2. Firstly, most coupled folding and binding events involve relatively short amphipathic motifs^39^. Secondly, IDPs show a unique preference to expose and use their hydrophobic residues for interaction^40^. Maybe, the present work may also provide insights into interactions between IDPs and their physiological targets.

In conclusion, with a novel computer strategy combined with SMD simulation and flexible docking, we predicted the spacer-α6 complex models and found that spacer may favor a partially unfolded (extended) α6. From the present spacer-α6 complex model, almost all key identified residues in both α6 and spacer through mutation experiments were predicted, demonstrating a better reliability of both our models and the computer strategy described here. The present models do provide a possible binding mode between the spacer and the extended α6, but as a technical challenge, further experimental confirmation is required. The present work should be contributed to better understanding of force-regulated cleavage of A2 by ADMATS13 and developing of antithrombotic antibody drugs targeting at ADAMTS13 or vWF-A2 domain.

## Materials and Methods

**Figure 8:**
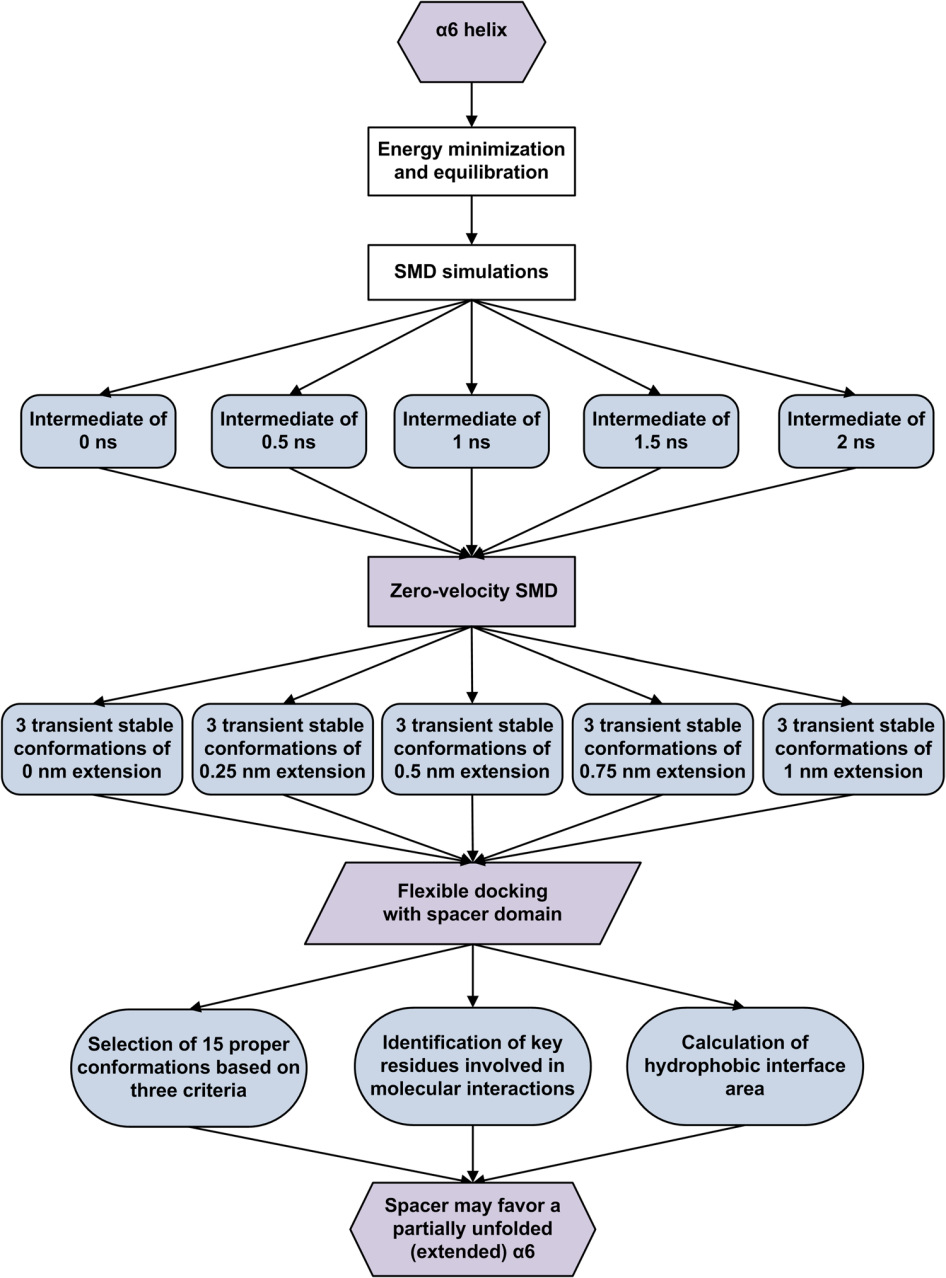
Ensemble workflow of computational procedure.

The strategy for computer prediction and theoretical analysis of the complex model of spacer bound with the stretched α6 helix was shown in the ensemble workflow of computational procedure (Fig. 8). All involved methods were described as below in detail.

### Molecular dynamics

The MD simulations were run with two software packages, the visual molecular dynamics (VMD) for visualization and modeling^41^, and the NAMD 2.6 program for steered MD simulations^42^. The structure of the α6 helix was obtained from the crystal structure of the A2 domain (PDB entry 3GXB) of vWF, and solvated with TIP3P water molecules in a rectangular box of 8.7 nm × 5.5 nm × 6 nm. The system was neutralized by adding 27 Na^+^ and 25 Cl^−^ ions. Periodical boundary condition and 2 fs time step were used to perform simulations with CHARMM22 all-atom protein force field^43^, along with CMAP correction for backbone, particle mesh Ewald algorithm for electrostatic interaction and a 1.3 nm cutoff for electrostatic and van der Waals interaction.

The system was subjected to an energy minimization of 15000 time steps at 0 ^◦^K twice, firstly with heavy or nonhydrogen atoms being fixed and secondly with all atoms free. Then, the system temperature was raised from 0 to 310 °K within 20 ps, followed by further 3 ns equilibration with controlled temperature and pressure in which the temperature was held at 310 ^◦^K using Langevin dynamics and the pressure was held at 1 atm by the Langevin piston method. An equilibrated conformation of α6 was obtained from the equilibrated system and taken as the initial conformation for steered MD simulations. The distance from the C_α_ atoms of the N-terminal residue Asp1653 to the C-terminal residue Arg1668 of α6 termed as peptide length was 2.089 nm.

### Steered Molecular Dynamics Simulations

SMD simulations were run on the equilibrated system with time step of 2 fs, where the N-terminal C_α_ atom was fixed while the C-terminal C_α_ atom was steered. The virtual spring with a spring constant of 700 pN/nm connected the dummy atom and the steered atom. Pulling was performed with a constant velocity of 0.5 nm/ns for 2 ns first, and then, along the pathway of the instantaneous time-course of extension of α6, five conformations at 0, 0.5, 1, 1.5 and 2 ns were selected respectively as the initial conformations for the subsequent SMD simulations of zero pulling velocity. Each run at zero pulling velocity lasted for 2 ns so that the violently stretched peptide was became relaxed through a structure relaxation process. Atomic coordinates were recorded every 2 ps. The time-courses of C_α_ root mean square deviation (RMSD) at zero pulling velocity were used to observe the transient stable or relaxed phases of the structure with different five initial stretched conformations. Three transient stable conformations almost with a same extension (the peptide length difference ≤ 0.04 nm) were randomly selected from the “pseudoplateaus” of the time-courses of RMSD simulated with zero pulling velocity for each initial conformation (Fig. 3) and taken as the starting structures for flexible dockings.

### Flexible docking

To model the spacer-α6 complex, flexible docking was performed via HADDOCK webserver (http://haddock.chem.uu.nl/services/HADDOCK/haddockserver-expert.html)^31^. The crystal structure of spacer (residues Ser556-Pro682; PDB code 3GHM) and each extended α6 conformation mentioned above were taken as starting structure. The Glycan NAG covalently linked to Asn614 of spacer was removed because the N-glycan was not required for the cleaving activity of ADAMTS13^44^. HADDOCK itself defined the ambiguous interaction restraints (AIRs) between active or passive residues in spacer and those in α6^32^. In spacer, the eight residues (Pro590, Leu591, Phe592, Leu637, Pro638, Arg660, Tyr661 and Try665) forming the exosite-3 were designated as active residues while their fourteen neighboring surface residues (Arg568, His588, Lys608, Met609, Ser610, Pro613, Asn614, Leu632, Glu634, Asp635, Glu664, Gly666, Asn667 and Leu668) were passive; and in α6, the hydrophobic residues (Leu1657, Pro1658, Ala1661, Pro1662, Leu1664, Val1665, Leu1666 and Gln1667), together with the residues (Asp1653, Glu1655 and Asp1663) whose mutations could affect cleavage, were regarded as active ones, while defining others (Phe1654, Thr1656, Arg1659, Glu1660 and Arg1668) as passive residues. Histidine protonation states in spacer were defined as HISD^31^. Both the N- and C-terminal in each of the spacer and the α6 were set to be neutral. All docking results for each starting structure of α6 were grouped into clusters, in which each was defined as an ensemble of at least four complex models with ligand interface RMSD smaller than 0.75 nm. The VMD program was used to generate the complex models and analyze the intermolecular salt bridges in binding site and the solvent accessible surface area (SASA) with a probe radius of 0.14 nm. Each complex model was subjected to a visual inspection through VMD, and only one was selected as proper model by following criteria (Supplementary Figs S1–5): (1) the orientation between N-termini of spacer and α6 must be correct to ensure the active center of ADAMTS13 to access the cleavage site, (2) the spacer and α6 bound with each other in a proper relative position whereby the hydrophobic residues in α6 faced the exosite-3 in spacer, (3) the model of α6 did not completely lose its helical structure integrity, and (4) a complex model with a lowest HADDOCK score.

### Data Availability

All data generated or analyzed during this study are included in this published article (and its Supplementary Information files).

## Electronic supplementary material

Supplementary information

## References

[1] Sadler JE (1998). Biochemistry and genetics of von Willebrand factor. Annu Rev Biochem.

[2] Lenting PJ, Christophe OD, Denis CV (2015). von Willebrand factor biosynthesis, secretion, and clearance: connecting the far ends. Blood.

[3] Dong JF (2005). Cleavage of ultra-large von Willebrand factor by ADAMTS-13 under flow conditions. J Thromb Haemost.

[4] Arya M (2002). Ultralarge multimers of von Willebrand factor form spontaneous high-strength bonds with the platelet glycoprotein Ib-IX complex: studies using optical tweezers. Blood.

[5] Zhang W (2015). Identification of a juxtamembrane mechanosensitive domain in the platelet mechanosensor glycoprotein Ib-IX complex. Blood.

[6] Dong JF (2003). ADAMTS-13 metalloprotease interacts with the endothelial cell-derived ultra-large von Willebrand factor. J Biol Chem.

[7] Sadler, J. E., Moake, J. L., Miyata, T. & George, J. N. Recent advances in thrombotic thrombocytopenic purpura. *Hematology Am Soc Hematol Educ Program*, 407–423, 10.1182/asheducation-2004.1.407 (2004). 10.1182/asheducation-2004.1.407 15561695

[8] Joly, B. S., Coppo, P. & Veyradier, A. Thrombotic Thrombocytopenic Purpura. *Blood*, 10.1182/blood-2016-10-709857 (2017). 10.1182/blood-2016-10-709857 28416507

[9] Manea M, Karpman D (2009). Molecular basis of ADAMTS13 dysfunction in thrombotic thrombocytopenic purpura. Pediatr Nephrol.

[10] Sadler JE (2005). New concepts in von Willebrand disease. Annu Rev Med.

[11] Zheng X (2001). Structure of von Willebrand factor-cleaving protease (ADAMTS13), a metalloprotease involved in thrombotic thrombocytopenic purpura. J Biol Chem.

[12] Zhang Q (2009). Structural specializations of A2, a force-sensing domain in the ultralarge vascular protein von Willebrand factor. Proc Natl Acad Sci USA.

[13] Wu T, Lin J, Cruz MA, Dong JF, Zhu C (2010). Force-induced cleavage of single VWFA1A2A3 tridomains by ADAMTS-13. Blood.

[14] Zhang X, Halvorsen K, Zhang CZ, Wong WP, Springer TA (2009). Mechanoenzymatic cleavage of the ultralarge vascular protein von Willebrand factor. Science.

[15] Lippok S (2016). Shear-Induced Unfolding and Enzymatic Cleavage of Full-Length VWF Multimers. Biophys J.

[16] Tsai HM (2004). Molecular mechanisms in thrombotic thrombocytopenic purpura. Semin Thromb Hemost.

[17] Zheng X, Nishio K, Majerus EM, Sadler JE (2003). Cleavage of von Willebrand factor requires the spacer domain of the metalloprotease ADAMTS13. J Biol Chem.

[18] Soejima K (2003). ADAMTS-13 cysteine-rich/spacer domains are functionally essential for von Willebrand factor cleavage. Blood.

[19] Gao W, Anderson PJ, Majerus EM, Tuley EA, Sadler JE (2006). Exosite interactions contribute to tension-induced cleavage of von Willebrand factor by the antithrombotic ADAMTS13 metalloprotease. Proc Natl Acad Sci USA.

[20] Zheng XL (2015). ADAMTS13, lucky to have a hydrophobic pocket. Blood.

[21] Ai J, Smith P, Wang S, Zhang P, Zheng XL (2005). The proximal carboxyl-terminal domains of ADAMTS13 determine substrate specificity and are all required for cleavage of von Willebrand factor. J Biol Chem.

[22] Gao W, Anderson PJ, Sadler JE (2008). Extensive contacts between ADAMTS13 exosites and von Willebrand factor domain A2 contribute to substrate specificity. Blood.

[23] Akiyama M, Takeda S, Kokame K, Takagi J, Miyata T (2009). Crystal structures of the noncatalytic domains of ADAMTS13 reveal multiple discontinuous exosites for von Willebrand factor. Proc Natl Acad Sci USA.

[24] Wu JJ, Fujikawa K, McMullen BA, Chung DW (2006). Characterization of a core binding site for ADAMTS-13 in the A2 domain of von Willebrand factor. Proc Natl Acad Sci USA.

[25] Pos W (2010). An autoantibody epitope comprising residues R660, Y661, and Y665 in the ADAMTS13 spacer domain identifies a binding site for the A2 domain of VWF. Blood.

[26] Bonvin AM (2006). Flexible protein-protein docking. Curr Opin Struct Biol.

[27] Smith GR, Fitzjohn PW, Page CS, Bates PA (2005). Incorporation of flexibility into rigid-body docking: applications in rounds 3-5 of CAPRI. Proteins.

[28] van Dijk AD (2005). Data-driven docking: HADDOCK’s adventures in CAPRI. Proteins.

[29] Crawley JT, de Groot R, Xiang Y, Luken BM, Lane DA (2011). Unraveling the scissile bond: how ADAMTS13 recognizes and cleaves von Willebrand factor. Blood.

[30] Ying J, Ling Y, Westfield LA, Sadler JE, Shao JY (2010). Unfolding the A2 domain of von Willebrand factor with the optical trap. Biophys J.

[31] de Vries SJ, van Dijk M, Bonvin AM (2010). The HADDOCK web server for data-driven biomolecular docking. Nat Protoc.

[32] de Vries SJ (2007). HADDOCK versus HADDOCK: new features and performance of HADDOCK2.0 on the CAPRI targets. Proteins.

[33] Zhu H, Domingues FS, Sommer I, Lengauer T (2006). NOXclass: prediction of protein-protein interaction types. BMC Bioinformatics.

[34] Tina KG, Bhadra R, Srinivasan N (2007). PIC: Protein Interactions Calculator. Nucleic Acids Res.

[35] Jin SY, Skipwith CG, Zheng XL (2010). Amino acid residues Arg(659), Arg(660), and Tyr(661) in the spacer domain of ADAMTS13 are critical for cleavage of von Willebrand factor. Blood.

[36] Di Stasio E (2008). Mechanistic studies on ADAMTS13 catalysis. Biophys J.

[37] Shim K, Anderson PJ, Tuley EA, Wiswall E, Sadler JE (2008). Platelet-VWF complexes are preferred substrates of ADAMTS13 under fluid shear stress. Blood.

[38] Tsai HM (1997). Proteolytic cleavage of recombinant type 2A von Willebrand factor mutants R834W and R834Q: inhibition by doxycycline and by monoclonal antibody VP-1. Blood.

[39] Wright PE, Dyson HJ (2009). Linking folding and binding. Curr Opin Struct Biol.

[40] Meszaros B, Tompa P, Simon I, Dosztanyi Z (2007). Molecular principles of the interactions of disordered proteins. J Mol Biol.

[41] Humphrey W, Dalke A, Schulten K (1996). VMD: visual molecular dynamics. J Mol Graph.

[42] Phillips JC (2005). Scalable molecular dynamics with NAMD. J Comput Chem.

[43] MacKerell AD (1998). All-atom empirical potential for molecular modeling and dynamics studies of proteins. J Phys Chem B.

[44] Zhou W, Tsai HM (2009). N-Glycans of ADAMTS13 modulate its secretion and von Willebrand factor cleaving activity. Blood.

