# Supplementary material for: Prediction of spacer-α6 complex: a novel insight into binding of ADAMTS13 with A2 domain of von Willebrand factor under forces
Source: Sci Rep. 2018 Apr 10;8:5791. doi: 10.1038/s41598-018-24212-6 (PMC5893608; doi:10.1038/s41598-018-24212-6)
Supplement: Supplementary file 1 — Supplementary information [file 41598_2018_24212_MOESM1_ESM.doc]

**Supplementary information**

**Prediction of spacer-α6 complex: a novel insight into binding of ADAMTS13 with A2 domain of von Willebrand factor under forces**

Xiang Fang, Jiangguo Lin, Ying Fang, Jianhua Wu

**SUPPLEMENTAL TABLES**

**Table S1. Selection of the complex model at the 0 nm extension of α6.**

**Table S2. Selection of the complex model at the 0.25 nm extension of α6.**

**Table S3. Selection of the complex model at the 0.5 nm extension of α6.**

**Table S4. Selection of the complex model at the 0.75 nm extension of α6.**

**Table S5. Selection of the complex model at the 1 nm extension of α6.**

**Table S1. Selection of the complex model at the 0 nm extension of α6.**

| **Complex models^*^** | **Relative orientation^†^** | **Relative position^‡^** | **Score^§^** | **Structural integrity^¶^** | **Selected model** |
| --- | --- | --- | --- | --- | --- |
| Cluster 2_1* | × |  | -72.288 | 80% |  |
| Cluster 2_2 | × |  | -71.593 | 80% |  |
| Cluster 2_3 | × |  | -70.259 | 100% |  |
| Cluster 2_4 | × |  | -67.446 | 80% |  |
| Cluster 1_1 | √ | + + + + + | -73.906 | 100% | Yes |
| Cluster 1_2 | √ | + + + + | -68.929 | 60% |  |
| Cluster 1_3 | √ | + + + + | -67.278 | 80% |  |
| Cluster 1_4 | √ | + + + + | -66.759 | 80% |  |
| Cluster 3_1 | × |  | -69.856 | 100% |  |
| Cluster 3_2 | × |  | -66.563 | 80% |  |
| Cluster 3_3 | × |  | -63.482 | 80% |  |
| Cluster 3_4 | × |  | -63.274 | 80% |  |
| Cluster 6_1 | × |  | -78.640 | 80% |  |
| Cluster 6_2 | × |  | -60.695 | 100% |  |
| Cluster 6_3 | × |  | -56.608 | 80% |  |
| Cluster 6_4 | × |  | -52.074 | 60% |  |
| Cluster 5-1 | × |  | -65.150 | 80% |  |
| Cluster 5_2 | × |  | -61.385 | 60% |  |
| Cluster 5_3 | × |  | -60.341 | 80% |  |
| Cluster 5_4 | × |  | -54.619 | 60% |  |
| Cluster 4_1 | √ | + + | -67.088 | 100% |  |
| Cluster 4_2 | √ | + + | -58.225 | 80% |  |
| Cluster 4_3 | √ | + + | -51.468 | 100% |  |
| Cluster 4_4 | √ | + + + | -42.218 | 60% |  |
| Cluster 8_1 | × |  | -76.544 | 100% |  |
| Cluster 8_2 | × |  | -50.004 | 80% |  |
| Cluster 8_3 | × |  | -43.724 | 80% |  |
| Cluster 8_4 | × |  | -18.336 | 100% |  |
| Cluster 7_1 | × |  | -49.213 | 80% |  |
| Cluster 7_2 | × |  | -48.100 | 100% |  |
| Cluster 7_3 | × |  | -36.626 | 60% |  |
| Cluster 7_4 | × |  | -36.298 | 80% |  |

**^*^**the top four models of each cluster; **^†^**relative orientation between spacer and α6; **^‡^**relative position between spacer and α6, i.e., the extent of contact between their hydrophobic surfaces; **^§^**HADDOCK score of each model; **^¶^**structural integrity of α6.

*Cluster 2_1 indicates the first model of cluster 2.

Table S2. Selection of the complex model at the 0.25 nm extension of α6.

| **Complex models** | **Relative orientation** | **Relative position** | **Score** | **structural integrity** | **Selected model** |
| --- | --- | --- | --- | --- | --- |
| Cluster 1_1 | √ | + + + | -78.855 | 60% |  |
| Cluster 1_2 | √ | + + + + + | -74.746 | 100% | Yes |
| Cluster 1_3 | √ | + + + + + | -71.386 | 80% |  |
| Cluster 1_4 | √ | + + + | -71.127 | 60% |  |
| Cluster 2_1 | × |  | -64.488 | 80% |  |
| Cluster 2_2 | × |  | -63.976 | 60% |  |
| Cluster 2_3 | × |  | -60.407 | 60% |  |
| Cluster 2_4 | × |  | -60.153 | 80% |  |
| Cluster 3_1 | × |  | -60.807 | 100% |  |
| Cluster 3_2 | × |  | -59.333 | 100% |  |
| Cluster 3_3 | × |  | -57.129 | 80% |  |
| Cluster 3_4 | × |  | -55.427 | 80% |  |
| Cluster 4_1 | √ | + + | -63.100 | 60% |  |
| Cluster 4_2 | √ | + | -55.306 | 80% |  |
| Cluster 4_3 | √ | + + | -49.478 | 80% |  |
| Cluster 4_4 | √ | + + + | -48.310 | 80% |  |
| Cluster 5-1 | √ | + + | -43.132 | 80% |  |
| Cluster 5_2 | √ | + + | -41.581 | 80% |  |
| Cluster 5_3 | √ | + + | -33.934 | 80% |  |
| Cluster 5_4 | √ | + + + | -24.930 | 80% |  |
| Cluster 6_1 | √ | + + | -55.911 | 80% |  |
| Cluster 6_2 | √ | + + | -49.604 | 80% |  |
| Cluster 6_3 | √ | + + | -22.632 | 0% |  |
| Cluster 6_4 | √ | + + | -6.263 | 80% |  |

Table S3. Selection of the complex model at the 0.5 nm extension of α6.

| **Complex models** | **Relative orientation** | **Relative position** | **Score** | **structural integrity** | **Selected model** |
| --- | --- | --- | --- | --- | --- |
| Cluster 4_1 | × |  | -79.498 | 60% |  |
| Cluster 4_2 | × |  | -77.671 | 80% |  |
| Cluster 4_3 | × |  | - 76.807 | 60% |  |
| Cluster 4_4 | × |  | -75.631 | 60% |  |
| Cluster 1_1 | √ | + + | -68.859 | 60% |  |
| Cluster 1_2 | √ | + + + + + | -68.099 | 80% | Yes |
| Cluster 1_3 | √ | + + + | -65.365 | 60% |  |
| Cluster 1_4 | √ | + + | -65.262 | 80% |  |
| Cluster 2_1 | √ | + + + + | -70.180 | 80% |  |
| Cluster 2_2 | √ | + + + + | -66.781 | 60% |  |
| Cluster 2_3 | √ | + + | -65.668 | 80% |  |
| Cluster 2_4 | √ | + + + + | -64.601 | 40% |  |
| Cluster 3_1 | × |  | -65.505 | 60% |  |
| Cluster 3_2 | × |  | -64.429 | 60% |  |
| Cluster 3_3 | × |  | -64.352 | 60% |  |
| Cluster 3_4 | × |  | -64.015 | 40% |  |
| Cluster 8_1 | × |  | -64.502 | 40% |  |
| Cluster 8_2 | × |  | -54.981 | 60% |  |
| Cluster 8_3 | × |  | -53.585 | 80% |  |
| Cluster 8_4 | × |  | -41.560 | 80% |  |
| Cluster 5_1 | √ | + + | -61.787 | 100% |  |
| Cluster 5_2 | √ | + + | -51.664 | 80% |  |
| Cluster 5_3 | √ | + + | -49.059 | 100% |  |
| Cluster 5_4 | √ | + + + | -47.958 | 60% |  |
| Cluster 7_1 | × |  | -71.739 | 80% |  |
| Cluster 7_2 | × |  | -60.949 | 60% |  |
| Cluster 7_3 | × |  | -37.615 | 80% |  |
| Cluster 7_4 | × |  | -34.049 | 100% |  |
| Cluster 6_1 | × |  | -47.841 | 60% |  |
| Cluster 6_2 | × |  | -47.820 | 40% |  |
| Cluster 6_3 | × |  | -47.082 | 60% |  |
| Cluster 6_4 | × |  | -37.382 | 60% |  |

Table S4. Selection of the complex model at the 0.75 nm extension of α6.

| **Complex models** | **Relative orientation** | **Relative position** | **Score** | **structural integrity** | **Selected model** |
| --- | --- | --- | --- | --- | --- |
| Cluster 4_1 | × |  | -75.254 | 80% |  |
| Cluster 4_2 | × |  | -74.775 | 80% |  |
| Cluster 4_3 | × |  | -70.811 | 80% |  |
| Cluster 4_4 | × |  | -66.421 | 100% |  |
| Cluster 3_1 | × |  | -72.742 | 100% |  |
| Cluster 3_2 | × |  | -71.017 | 40% |  |
| Cluster 3_3 | × |  | -68.363 | 80% |  |
| Cluster 3_4 | × |  | -65.000 | 60% |  |
| Cluster 1_1 | √ | + + + | -68.051 | 60% |  |
| Cluster 1_2 | × |  | -64.519 | 80% |  |
| Cluster 1_3 | √ | + + + + | -63.071 | 60% |  |
| Cluster 1_4 | √ | + + + + + | -62.578 | 80% | Yes |
| Cluster 5_1 | × |  | -69.912 | 20% |  |
| Cluster 5_2 | × |  | -61.857 | 80% |  |
| Cluster 5_3 | × |  | -61.346 | 60% |  |
| Cluster 5_4 | × |  | -61.037 | 80% |  |
| Cluster 6_1 | × |  | -70.558 | 80% |  |
| Cluster 6_2 | × |  | -59.765 | 80% |  |
| Cluster 6_3 | × |  | -38.960 | 20% |  |
| Cluster 6_4 | × |  | -38.515 | 60% |  |
| Cluster 2_1 | √ | + + + | -52.424 | 60% |  |
| Cluster 2_2 | √ | + | -47.779 | 80% |  |
| Cluster 2_3 | √ | + + | -44.071 | 40% |  |
| Cluster 2_4 | √ | + + | -43.791 | 40% |  |

Table S5. Selection of the complex model at the 1 nm extension of α6.

| **Complex models** | **Relative orientation** | **Relative position** | **Score** | **structural integrity** | **Selected model** |
| --- | --- | --- | --- | --- | --- |
| Cluster 1_1 | × |  | -90.978 | 80% |  |
| Cluster 1_2 | × |  | -87.780 | 80% |  |
| Cluster 1_3 | × |  | -87.501 | 80% |  |
| Cluster 1_4 | × |  | -87.027 | 80% |  |
| Cluster 6_1 | × |  | -73.897 | 60% |  |
| Cluster 6_2 | × |  | -67.865 | 20% |  |
| Cluster 6_3 | × |  | -66.266 | 60% |  |
| Cluster 6_4 | √ | + + | -53.120 | 80% |  |
| Cluster 3_1 | × |  | -75.376 | 60% |  |
| Cluster 3_2 | √ | + + | -64.409 | 60% |  |
| Cluster 3_3 | √ | + + + + | -58.838 | 80% | Yes |
| Cluster 3_4 | × |  | -51.627 | 0% |  |
| Cluster 2_1 | × |  | -65.168 | 60% |  |
| Cluster 2_2 | × |  | -62.350 | 60% |  |
| Cluster 2_3 | × |  | -58.722 | 80% |  |
| Cluster 2_4 | × |  | -58.715 | 60% |  |
| Cluster 4_1 | × |  | -63.564 | 40% |  |
| Cluster 4_2 | × |  | -60.981 | 20% |  |
| Cluster 4_3 | × |  | -57.281 | 40% |  |
| Cluster 4_4 | × |  | -55.687 | 80% |  |
| Cluster 5_1 | × |  | -52.424 | 60% |  |
| Cluster 5_2 | × |  | -47.779 | 40% |  |
| Cluster 5_3 | × |  | -44.071 | 60% |  |
| Cluster 5_4 | × |  | -43.791 | 60% |  |
